# Vibrational Spectroscopic Analyses and Imaging of the Early Middle Ages Hemp Bast Fibres Recovered from Lake Sediments

**DOI:** 10.3390/molecules26051314

**Published:** 2021-03-01

**Authors:** Grzegorz Kalisz, Barbara Gieroba, Olga Chrobak, Magdalena Suchora, Agata L. Starosta, Anna Sroka-Bartnicka

**Affiliations:** 1 Department of Biopharmacy, Medical University of Lublin, ul. Chodzki 4a, 20-093 Lublin, Poland; grkalisz@gmail.com (G.K.); barbaragieroba@umlub.pl (B.G.); 2 Department of Molecular Biology, Institute of Biological Sciences, Maria Curie-Sklodowska University, ul. Akademicka 19, 20-400 Lublin, Poland; olga.chrobak@poczta.umcs.lublin.pl; 3 Laboratory of Gene Expression, ECOTECH-Complex, Maria Curie-Sklodowska University, ul. Gleboka 39, 20-612 Lublin, Poland; magdalena.suchora@umcs.pl; 4 Department of Geomorphology and Paleogeography, Institute of Earth and Environmental Sciences, Maria Curie-Sklodowska University, Poland, al. Krasnicka 2d, 20-711 Lublin, Poland; 5 Department of Genetics and Microbiology, Institute of Microbiology and Biotechnology, Maria Curie-Sklodowska University, ul. Akademicka 19, 20-033 Lublin, Poland

**Keywords:** hemp (*Cannabis sativa* L.), natural fibres, retting, polysaccharides, FTIR imaging, ancient plant remains

## Abstract

Fourier Transform Infrared (FT-IR) spectroscopy and imaging combined with hierarchical cluster analysis (HCA) was applied to analyse biochemical properties of Early Middle Ages hemp (*Cannabis sativa* L.) bast fibres collected from lake bottom sediment of lake Słone. The examined plant macrofossil material constitutes residues of the hemp retting process that took place in the 7th–8th century. By comparison of three samples: untreated isolated bast fibres, and fibres incubated overnight at 4 and 37 °C, we were able to mimic the retting conditions. Using FT-IR qualitative and semi-quantitative assessment of the primary polysaccharides content, total protein content, and their spatial distribution was performed within the hemp fibres. The concentration of cellulose remained vastly unchanged, while the concentration of lignin and pectin was the highest in the untreated sample. The spatial distributions of compounds were heterogeneous in the untreated and 4 °C-incubated samples, and homogenous in the specimen processed at 37 °C. Interestingly, a higher amide content was detected in the latter sample indicating the highest degree of enzymatic degradation. In this study, we show that the spectroscopic methods allow for a non-destructive evaluation of biochemical composition of plant fibres without preparation, which can be an appropriate approach for studying ancient plant remains.

## 1. Introduction

Situated in SE Poland, lake Słone (51°18′16″ N, 23°21′55″ E) is an extensively studied paleolimnological site with a well-documented human impact on the natural environment of the region during the past 13,000-years of Late Glacial and Holocene [1,2]. The most prominent turning point in the lake’s history was the Early-Medieval episode of its use as hemp (*Cannabis sativa* L.) rettery, which is documented by the results of sedimentary pollen analysis [1]. Hemp fibres have been commonly used by humans living in different parts of the world for at least 6000-years due to their unique properties, such as exquisite fibre strength and exceptional length. They were utilized to make ropes, sails, textiles and even paper [3]. The contemporary renaissance of hemp use has been observed in the last decade and is mostly due to very good technical parameters including superior stiffness and low density as well as cost-efficiency and environmental friendliness of hemp fibre products resulting from high biodegradability [4].

In the early Middle Ages, hemp fibre was obtained by retting the stems in natural waterbodies including lakes or ponds with an example of such early Slav’s rettery being lake Słone. In the process of retting of the bast fibre plants, the cellulose-rich fibres of the sclerenchyma are separated from the non-cellulosic plant components and the pectic polysaccharidic lamella that surrounds the cellulose bundles is broken down. This is an enzymatic microbial process, mostly dependent on the pectinolytic capabilities of the retting microbial community [5].

The hemp bast fibres are extraxylary sclerenchymal cells of the gelatinous type, characterised by thick cellulosic cell wall [6]. Elementary components in the hierarchical structure of hemp fibres are microfibrils with a diameter of 5–10 nm and length ranging from 100 nm to several micrometres, which are composed mainly of celluloses accompanied by hemicelluloses. These basic fibres are fixed together with pectins and to a lesser extent with lignins, forming a specific pectin-lignin matrix [3,7]. Cellulose is a β-D-glucose linear polymer containing anhydro-D-glucopyranose units that are linked together with (1,4)-β-glycosidic bonds [8], while hemicellulose is a heterogenous polysaccharide polymer composed of highly branched low-molecular-weight homo- and heteropolymer sugars, like D-xylopyranose, D-galactopyranose, D-glucopyranose, L-arabinofuranose, D-mannopyranose, D-glucopyranosyluronic acid and D-galactopyranosyluronic acid with slight admixture of other sugars [9,10]. Pectins are galacturonic acid rich heteropolysaccharides comprised of homogalacturonan, rhamnogalacturonan I and the glycosylated, methylated, and acetylated galacturonans, rhamnogalacturonan II (RG-II) and xylogalacturonan (XGA) units linked via (1,4)-α-glycosidic bonds in the backbone [11]. In turn, lignins form a complex, high-molecular-weight, insoluble, cross-linked phenolic polymer with amorphous, non-crystalline structure located in the cell wall between cellulose and hemicellulose polymers. They include numerous methoxylated derivatives of benzene (phenylpropanoid alcohols-monolignols), especially coumaryl, coniferyl and sinapyl [12]. Hemp fibres also contain small amounts of waxes and pigments (1.2–6.2%), residual ashes (0.8%), moisture, and other organic substances including proteins [13,14].

Fourier Transform Infrared (FT-IR) spectroscopy has become a useful and versatile tool for studying a variety of plant tissues. It allows obtaining biochemical information on the composition and structure of the components as a result of the identification of the major functional groups and chemical bonds in the biological samples [15]. Coupled with microscopy, it enables tracking the distribution of particular constituents within the specimen [16,17]. It has a significant advantage over other microscopic methods applied on their own, such as scanning electron microscopy (SEM), transmission electron microscopy (TEM), atomic force microscopy (AFM) and optical microscopy, which provide merely morphological data of the studied material [18]. Furthermore, FT-IR spectroscopy can be applied for analysing variations in the content of the primary organic compounds, both between and within the same species subjected to various chemical conditions [19]. It was reported that FT-IR spectroscopy has been so far successfully used for plant fibres studies [20], including hemp fibres [3,4]. Despite limitations in the acquisition and interpretation of plant samples spectra resulting from biochemical complexity, signal overlapping and the impact of particle size on FT-IR absorbance, latest progress in the measurement methods enabled an increase in the resolution of in situ compositional heterogeneity and made it possible to conduct a semi-quantitative analysis using this technique [21]. It is worth emphasizing that benefits in favour of the choice of the infrared measurements include non-invasiveness, data collection speed and reproducibility, and ease of both the maintenance and use of the device [15]. FT-IR allows collecting sample spectra with very good signal-to-noise ratio (S/N) compared to other techniques, achieving high quality spectra. Additionally, FT-IR imaging does not require the use of dyes and labelling methods for different chemical components [22].

The objective of this work was to qualitatively and semi-quantitatively evaluate, with the application of Fourier Transform Infrared spectroscopy and imaging as well as multivariate chemometric approach, the content of pectin, lignin and cellulose in ancient hemp bast fibres remains dating back to the 7th–8th century and derived from lake Słone. The samples were subjected to overnight enzymatic degradation carried out by the sedimentary microorganisms deposited on the fibre residues. The sediment samples were demonstrated to contain live microorganisms as verified with microbiological and molecular biology methods (data not shown). The enzymatic digestion was performed at two different temperatures: 4 and 37 °C. The temperature of 4 °C was selected to mimic the conditions of the near-bottom water layer of the stratified lake. The higher temperature was chosen as it is optimal for most microorganisms and ensures fast and non-selective microbial growth. As a control, hemp fibre remains freshly extracted from the frozen sediment material were used. The applied spectroscopic approach provided an opportunity to study the biochemical composition of ancient hemp fibre remains and component distribution within the samples without the need for complicated preparation procedures and staining. Moreover, it allowed to monitor the microbial activity revealed by chemical composition changes of the studied material, indicative of enzymatic hydrolysis of the primary plant polysaccharides.

## 2. Results

### 2.1. FT-IR Spectroscopy

**Figure 1:**
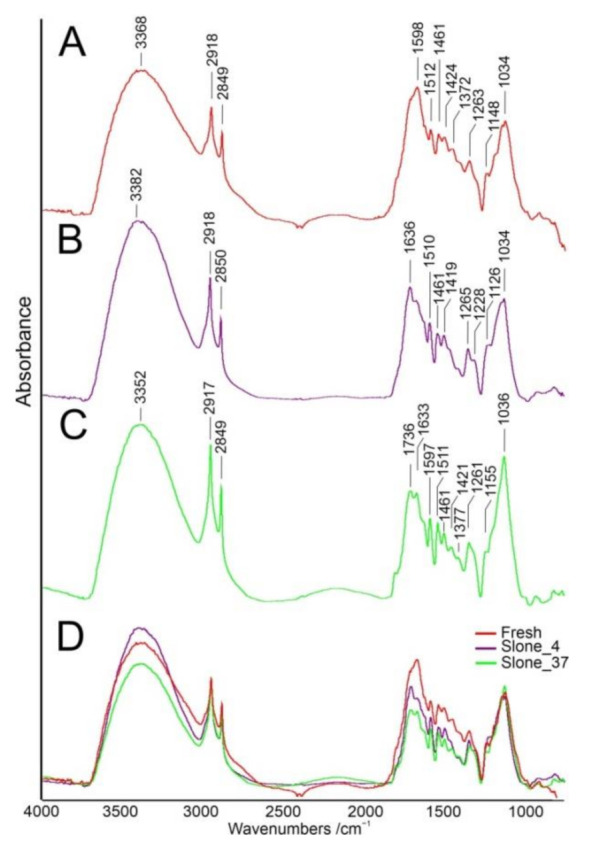
Averaged FT-IR spectra of Fresh (**A**), Slone_4 (**B**) and Slone_37 (**C**) samples, (**D**) overlaid spectra. All spectra are normalized to the 2918 cm^−1^ band.

FT-IR spectra of the investigated samples show differences in the “fingerprint” region containing cellulose, lignin and pectin bands (Figure 1). The observed bands are shown in Table 1 with the assignments to plant polysaccharides according to Zimniewska et al. [23].

In the Fresh sample, a weak absorbance band at 894 cm^−1^ was recorded and assigned to ν(C-O-C) in plane, symmetric vibrations, characteristic for β-glycosidic bonds. Such bonds appear in cellulose with (1,4)-β-glycosidic linkages and in hemicellulose and pectin with (1,4)-β and (1,3)-β-glycosidic linkages respectively [23]. Spectral region of 1160–1120 cm^−1^ is ascribed to the absorption of glycosidic bonds with a shift depending on the conditions which the cellulose fibres were subjected to. According to Fan et al., under moist conditions with increased pressure cellulosic material is subjected to spectral deformations corresponding to crystalline-to-amorphous shifts without any molecular changes, with particular sensitivity of 1420 and 894 cm^−1^ bands [20]. Such a change is attributed to the cell wall dislocation within hemp fibres. Similarly, in dislocated regions bands at ~1370 cm^−1^ almost disappear, what is observed in sample Slone_4 (Figure 1). This indicates a loss of lignin in the studied sample, resulting in the lower absorbance wavenumbers [20]. Since lignin is a polymer derived from phenolic alcohols, it has characteristic spectral vibrations at 1595–1593 cm^−1^ range arising from aromatic C=C bond ring. To evaluate the pectins content, pectin-characteristic bands were chosen according to Synytsya et al. [28]. Spectral regions of C-H stretching at 3600–3200 cm^−1^ and O-H stretching at 2600 cm^−1^ in carboxyl group were not considered due to the overlapping with O-H stretching vibration of hydroxyls and water. Thus, a spectral band at 1735 cm^−1^ was selected as indicative of pectins. Comparison of the calculated ratios for pectins confirmed higher concentration in sample Slone_37 compared to Slone_4 and the highest concentration in sample Fresh. Similar observation was made at 1419 cm^−1^ band (Figure 1).

To compare the relative concentrations of carbohydrates in hemp fibres, three ratios were calculated. In accordance with Garside and Wyeth [24], the relative intensities were measured at 1735, 1595 and 1105 cm^−1^ wavenumbers for pectin, lignin and cellulose respectively. The intensity of C-H stretching at 2900 cm^−1^, corresponding to a group of aliphatic chains of carbohydrates, proteins and lipids was selected for the total organic material in the fibre residues according to the abovementioned article [24]. The following ratios were calculated on the basis of the peak height:

Lignin R_1_ = I_1595_/I_2900_ (1)

Cellulose R_2_ = I_1105_/I_2900_ (2)

Pectin R_3_ = I_1735/_I_2900_ (3)

**Figure 2:**
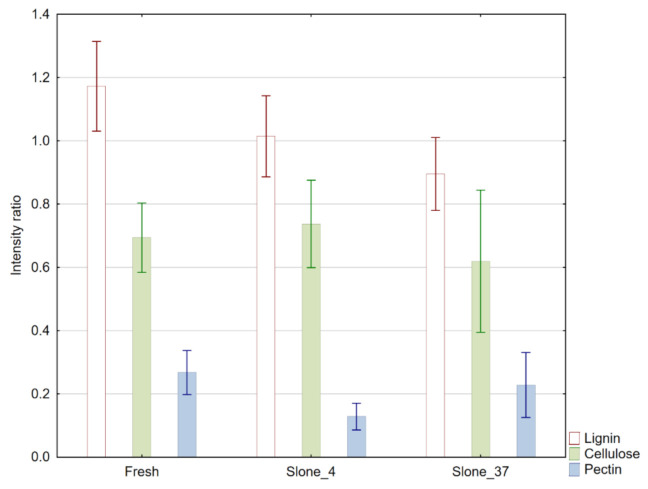
Semi-quantitative representation of pectin (blue), lignin (brown) and cellulose (green) in samples Fresh, Slone_4 and Slone_37. The relative quantities are based on the calculated intensity ratios R_1,_ R_2_ and R_3_ for each plant polysaccharide. Error bars represent standard deviation of the absorbance intensity ratio.

Absorbance intensity ratios were calculated for 10 randomly selected measurement points for each sample. Results were averaged and presented in Figure 2 with standard deviation as error bars and in Table A1 in Appendix A. Comparison of the results revealed similar concentrations of cellulose in all samples, with the highest amount in Slone_4. Lignin concentration was the highest in Fresh sample, and gradually decreasing in Slone_4 and further in Slone_37, while the highest concentration of pectins was found in Fresh and the lowest in Slone_4.

**Figure 3:**
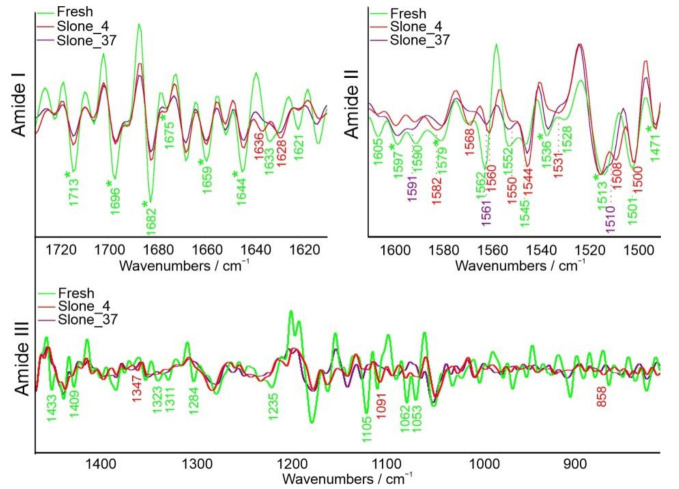
Second derivative of FT-IR spectra of amide I (1730–1610 cm^−1^), amide II (1610–1490 cm^−1^) and amide III (1450–800 cm^−1^) overlapping partially with polysaccharides (1200–800 cm^−1^). Asterisk (*) indicates the same value recorded for each spectrum.

Due to the revealed changes in polysaccharides concentrations in the studied samples, as well as enzymatic bases of the hemp retting process, the protein content was also examined. More detailed information on the chemical composition differences between the samples for both proteins and polysaccharides was provided with a second order derivative spectra. Three regions were chosen: amide I, amide II and amide III (Figure 3), considered useful in monitoring variations in intensities of absorbance which may resolve broad, overlapping bands, increasing the accuracy of analysis. Second derivative shifts in the range assigned to the amide I and II are caused by changes in the secondary structure of proteins and hydrogen-bonding. The amide III region was considered less helpful in the analysis of protein secondary structures composition due to the range of 1200–800 cm^−1^, partially overlapping with spectra containing bands assigned to water, lipids and carbohydrates. However, it did provide additional data about the glycosidic linkages of the polysaccharides chains.

In amide I most significant differences were observed between samples Fresh and Slone_4; sample Slone_37 was similar to Slone_4 (Figure 3). In case of sample Fresh, 1675 cm^−1^ band was more pronounced and new bands at 1621 and 1633 cm^−1^ appeared. Spectra of samples Slone_4 and Slone_37 also gained a new band at 1628 cm^−1^. In the spectrum of sample Slone_4, a band at 1636 cm^−1^ emerged as well. All bands are correlated to changes in β-sheet structures [29]. Second derivative of spectra in amide II region is relatively similar in each tested sample with only minor shifts. Few shifts can also be observed in amide III region. Additionally, bands present in sample Fresh at 1433, 1409, 1323, 1311, and 1284 cm^−1^ disappeared in the processed samples during hydrolysis at both 4 and 37 °C. All of these may indicate changes in protein content composition in samples Slone_4 and Slone_37—the amount of proteins with α-helices and random coil secondary structures decreased compared to sample Fresh [30,31,32]. Additional bands in 1245–1225 cm^−1^ range observed in Slone_4 sample indicate a higher content of β-sheets [31]. The microbial hydrolysis at a higher temperature further led to the disappearance of the 1235 cm^−1^ band, suggesting decreasing amount of β-sheets in sample Slone_37 compared to Slone_4 (Figure 3). This may be due to the fact that during the hemp retting process bacteria synthesize new enzymes with predominantly β-sheets in their molecular arrangement. Such enzymes include for example pectinesterases and pectinases (consisting mainly of right-handed parallel β-sheets), whose increased production consequently leads to increased degradation of pectin, which indeed was the case in sample Slone_4 [33]. Moreover, in Slone_4 and Slone_37 the band at 834 cm^−1^ ascribed to ν(CC) of α-glycosidic linkage observed in Fresh sample, is shifted to 858 cm^−1^ and less pronounced, indicating most probably cleavage of these bonds resulting in pectin backbone breakdown [34].

### 2.2. FT-IR Spectroscopic Imaging

**Figure 4:**
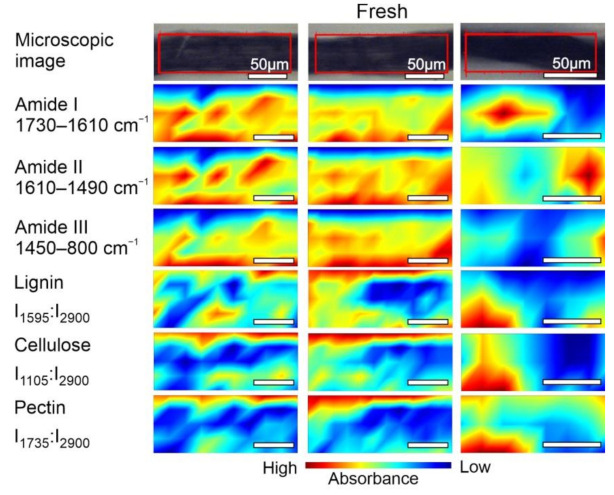
Microscopic images and FT-IR chemical maps of Fresh samples group. Three individual fibre cross sections are presented in columns. Rows represent maps of chemical distribution of amide I (1730–1610 cm^−1^), amide II (1610–1490 cm^−1^), amide III (1450–800 cm^−1^), lignin (I_1595_:I_2900_), cellulose (I_1105_:I_2900_) and pectin (I_1735_:I_2900_). White bars represent 50 µm.

**Figure 5:**
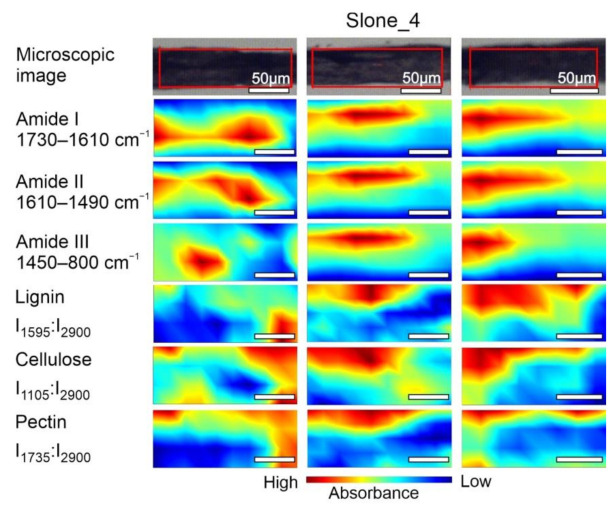
Microscopic images and FT-IR chemical maps of Slone_4 group. Three individual fibre cross sections presented in columns. In rows maps of chemical distribution of amide I (1730–1610 cm^−1^), amide II (1610–1490 cm^−1^), amide III (1450–800 cm^−1^), lignin (I_1595_:I_2900_), cellulose (I_1105_:I_2900_) and pectin (I_1735_:I_2900_). White bars represent 50 µm.

**Figure 6:**
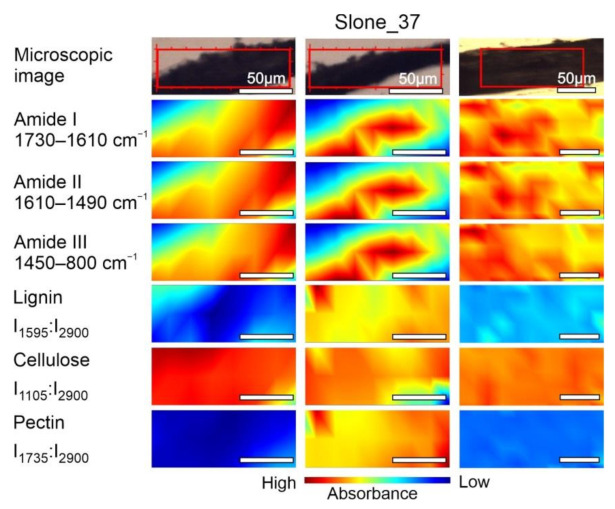
Microscopic images and FT-IR chemical maps of Slone_37 group. Three individual fibre cross sections presented in columns. In rows maps of chemical distribution of amide I (1730–1610 cm^−1^), amide II (1610–1490 cm^−1^), amide III (1450–800 cm^−1^), lignin (I_1595_:I_2900_), cellulose (I_1105_:I_2900_) and pectin (I_1735_:I_2900_). White bars represent 50 µm.

The chemical composition of samples Fresh, Slone_4 and Slone_37 was examined by mapping the fibres area as shown in Figure 4, Figure 5 and Figure 6. The concentration of compounds is associated with the intensity of particular, characteristic bands, based on the data in Table 1. Spatial distribution of the chosen compounds allowed to evaluate the homogeneity of distribution.

The spectroscopic imaging data shown in Figure 4 enabled comparison of the differences in the distribution of the selected compounds within Fresh sample. In order to compare the samples, the same analysis was performed for Slone_4 (Figure 5) and Slone_37 (Figure 6).

FT-IR microspectroscopical analysis revealed the diversified arrangement of amides and polysaccharides both in Fresh (Figure 4) and Slone_4 (Figure 5) specimen. The compound distribution in Slone_37 presented a slightly different pattern (Figure 6).

Considering the intensity of absorbance, the pectin and lignin content in the Slone_4 and Fresh samples is similar and the distributions of both polysaccharides are heterogenous in these two samples. This is very different in Slone_37 as the distributions of lignin and pectin ratios are more homogenous and the contents are lower in comparison to Fresh and Slone_4 samples. In case of cellulose ratio (I_1105_:I_2900_), in Slone_37 the cellulose content is the highest comparing to Fresh and Slone_4 and the spatial distribution shows much higher degree of homogeneity compared to samples Fresh and Slone_4. Thus, sample Slone_37 is characterised by the highest degree of spatial homogeneity regarding the three plant polysaccharides. Moreover, sample Slone_37 also presented a higher intensity of bands corresponding amides I, II and III suggesting a higher amide content. The high intensities of amide bands together with low intensities and homogeneous distributions of lignin and pectin, may indeed indicate more overall enzymatic degradation of the studied compounds in the sample Slone_37 compared to samples Slone_4 and Fresh which show heterogenous distribution of both amides and polysaccharides.

### 2.3. Hierarchical Cluster Analysis (HCA)

**Figure 7:**
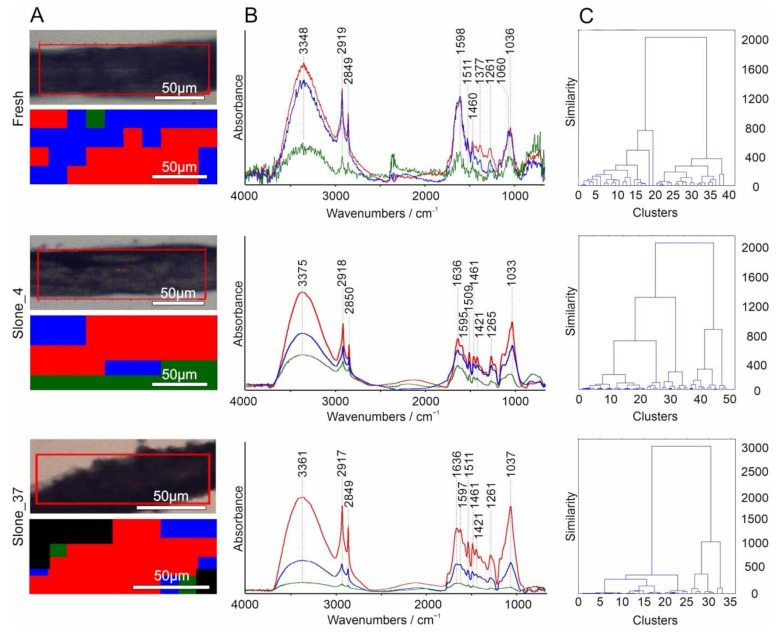
The Hierarchical cluster analysis using D-value distance and Ward’s algorithm of Fresh, Slone_4 and Slone_37 samples. (**A**) visible image with marked area of mapping and chemical image of three clusters, (**B**) averaged spectra from the three clusters, the colour in the map corresponds to the averaged spectrum from the cluster, and (**C**) dendrograms.

The application of hierarchical cluster analysis (HCA) for multivariate statistical evaluation with D-value and Ward’s algorithm markedly increased the information content of the FT-IR images and allowed to explore the degree of similarity between the hemp fibres samples. Chemometrics as a multidisciplinary field of study extracts essential data from extensive (bio)chemical sets of information with the application of diverse mathematical and statistical techniques. The Figure 7A presents a spatial distribution of spectra which were assembled into clusters based on their similarity. The averaged FT-IR spectra corresponding to the three clusters obtained from HCA analysis are presented in Figure 7B. The hierarchical cluster analysis dendrograms based of the FT-IR spectra of the complete spectral data set (Ward’s distance clustering algorithm) are shown in Figure 7C.

HCA analysis calculated with D-values and Ward’s algorithm (Figure 7) of Fresh, Slone_4 and Slone_37 samples was performed to characterize the comprehensiveness of spectral variations concerning the examined components and to explore the connectivity and hidden patterns within and between samples where the relationship of data and grouping were previously unclear. The result of HCA is usually presented in a dendrogram, which is a plot presenting in a tree form the organization and hierarchy of grouped data showing similar spectral characteristics and thus determining the structural characteristics of the variables among samples. The number and spatial clustering of the groups of spectra obtained using Ward’s algorithm presents the similarities and relationships for all spectra in the samples. In the sample Fresh, bands corresponding to pectin and lignin (spectral region of 1700–1500 cm^−1^) have higher absorbance compared to the bands in the spectral region of cellulose (1100–950 cm^−1^). In the sample Slone_4 all bands corresponding to the three polysaccharides have similar absorbances, whereas in the sample Slone_37 the bands in the spectrum range corresponding to cellulose (1100–9500 cm^−1^) have higher absorbance compared to lignin and pectin (spectrum range of 1700–1500 cm^−1^) (Figure 7B). This is reflected in the dendrograms of the three samples. Slone_37 differs from Fresh and Slone_4 by the emergence of three clusters at the level of 2000 compared to two clusters for samples Fresh and Slone_4 at the same level. Earlier separation of one of the clusters probably testifies to more intensive enzymatic degradation in this sample, resulting in two groups of spectra based on their chemical composition. The latest separation into three classes was observed in Fresh sample (at the level below 800), suggesting least intense chemical decomposition. Moreover, the four classes at 800 are present only in the Slone_4 sample. The variability in the biocomposition of the samples illustrated with the dendrograms also corresponds to different arrangement of components in chemical images and slight shifts in band positions evident in the average spectra.

## 3. Discussion

For centuries hemp fibres have been extensively used throughout the world for the production of everyday items. The remains of such items as well as of the bast fibre resulting from hemp retting process are rarely found near former human settlements [35] due to the susceptibility to degradation by microorganisms and low thermal stability [13] which resulted in only little material surviving to this day, mostly in the bottom sediments of water bodies used as retteries [36,37].

In this study, we investigated the process of polysaccharide degradation involved in retting of the hemp bast fibre remains at the temperatures of 4 °C and 37 °C at a laboratory scale. Water retting, which is largely led by anaerobic bacteria, is determined by the microbial culture composition, which in turn influences the enzymatic capabilities and different degradation rates of the plant polysaccharides. Usually, pectin degradation has the highest rate, followed by cellulose and lignin deterioration [38]. Due to the microbial nature of the process, water retting can be controlled by water temperature which determines bacterial growth rate [39]. The obtained results indeed demonstrated degradation of the primary plant polysaccharides in the samples Slone_4 and Slone_37 which could be attributed to different bacterial degradation processes depending on the retting temperature. Thus, FT-IR measurements allowed for simultaneous analyses of different polysaccharides in the limited amount of the ancient hemp fibres remains without damaging the plant material.

Pectin hydrolysis is central to the retting process as it loosens the fibre bundles from the stem tissues rendering pectinases most important enzymes in retting [40]. Pectinolytic enzymes are a group of heterogeneous enzymes that can be broadly divided into three groups: depolymerases (hydrolases and lyases), esterases, and protopectinases [41]. Pectin esterases catalyse deesterification of the methoxyl group of pectin, while hydrolases and lyases are involved in cleavage of the α-1,4-glycosidic bonds [42]. Interestingly, in our studies pectin concentration was higher in Slone_37 compared to Slone_4 possibly suggesting a higher pectinolytic activity at a lower temperature. Moreover, the degradation of pectin was also evidenced by changes in the protein secondary structures, strongly suggesting the production of pectin hydrolysing enzymes. This was in particular showed by the amide I and II bands as the increase in β-sheets in sample Slone_4 which may indicate increasing levels of different pectinolytic enzymes [43]. Degradation of plant polysaccharides was further showed with the polysaccharide contribution in the second order derivative course in amide III spectral range suggesting cleavage of the α-glycosidic bonds, which may imply hydrolysis to smaller subunits and/or monosaccharides with different molecular structures. The optimum temperature range for most of the microbial pectinases characterised in the literature is 30–50 °C [44] however, Magnusson and Svennerstedt (2007) [39] reported that high temperatures (over 45 °C) are detrimental to the pectinolytic processes during retting and no difference between 30 °C and 37.5 °C was described. At the same time, microorganisms producing high level of cold-active pectinolytic enzymes, acting at the temperatures of 4–5 °C, were isolated [45,46]. It should be noted however, that environmental strains unculturable under laboratory conditions may in fact have an important role in pectin hydrolysis during water retting. Hence, higher pectin degradation at low temperature, as seen in this study, was most probably due to specificity of the microbial community adapted to living conditions of this particular environmental niche—the bottom, hypolimnetic layer of a lake. In contrast to pectin, lignin level clearly decreased with an increasing temperature of incubation compared to the untreated material. Lignin-degrading enzymes are divided into two primary groups: lignin-modifying enzymes (LME) and lignin-degrading auxiliary (LDA) enzymes, with the latter necessary to conclude the degradation process [47]. The central process of lignin hydrolysis is enzymatic breakdown of the aromatic ring which requires oxygen (or reactive oxygen species) and thus, the complete lignin digestion cannot be performed under anaerobic conditions [38]. As lignin is a high molecular weight highly branched molecule, degradation process is relatively slow. Although enzymatic digestion was shown to be the most efficient approach to lignin degradation during retting, over-retting of lignin does not usually occur [48]. Hence, the effect of temperature on lignin content in the investigated samples might have been due to more favourable thermodynamic conditions of the enzymatic reactions carried out at a higher temperature rather than the enzymatic capabilities of the retting microbial culture.

As the ultimate goal of the retting process is removal of mostly pectins and lignins and recovery of the cellulosic fibres, cellulose degradation is an unwanted by-product of microbial action [48]. Lignin and pectin stereochemistry is irregular and less specific enzymes are required for their degradation whereas unbranched, very densely packed and crystalline microfibrils forming cellulose is highly resistant to enzymatic degradation. Many different oxidative “endo- and exo-acting” hydrolytic enzymes are needed for cellulose digestion [49] providing molecular basis for the retting process. However, over-retting of the cellulose fibres is not uncommon and is usually caused by inadequate (too long) retting times [48]. In this study, ancient hemp bast fibre remains were examined and because of the material fragility, minimal incubation times were applied. Such short digestion time indeed did not affect cellulose content of the fibres as only slight variations were detected in the amount of cellulose between the samples.

Based on our analysis it can be concluded that the most intensive enzymatic activity occurred in the Slone_37 sample as evidenced by homogenous distribution of polysaccharides and higher amide content. The differences in the secondary structures visible in the course of the second derivative in the amide bands presumably testify to the change in the composition of enzymes secreted by the bacteria involved in hemp retting. These enzymes are most likely responsible for degradation of the investigated plant polysaccharides. The higher enzymatic activity at 37 °C was most probably a result of higher microbial growth and/or activity. However, in the sample incubated at 4 °C the examined biochemical composition also suggests adequate retting processes. In both conditions the content and distribution of plant polysaccharides changed as compared to the undigested sample suggesting microbial enzymatic activity, in particular of pectinases and lignin-modifying enzymes. Thus, it can be concluded that hemp fibres dating back about 1200-years were digested by the microorganisms present in the sediment samples. They preserved the ability to grow and produce active, fully functioning enzymes in varying temperatures.

FT-IR microspectroscopical and chemometric analyses performed here provided information about the (bio)chemical composition and distribution of the individual compounds, such as different plant polysaccharides, in the ancient remains of hemp bast fibres [50,51]. Despite the numerous advantages of this method, data analysis and interpretation may be a very challenging task. In the “fingerprint” region (1500–500 cm^−1^) of the spectra, which includes the cellulose, pectin, lignin, and amides ranges, many overlapping bands were observed [24]. To investigate the contribution of the underlying bands, the second derivatives were determined [52] whereas in order to analyse polysaccharides content statistically, the absorbance intensity ratios were calculated. These spectral operations resulted in a more complete view of qualitative and semi-quantitative composition of hemp fibres, providing a detailed analysis [24].

The chemometric analysis of the obtained clusters in dendrograms presented in the Figure 7C further clarifies the homogeneity of the samples. Basing on the dendrograms the most homogenic sample is Slone 37 > Fresh > Slone 4. According to the averaged spectra of the three clusters (Figure 7B) in the Fresh sample the most intense bands are these corresponding to pectin and lignin in the range of 1700–1500 cm^−1^. In the Slone_37 sample, the bands from cellulose (range of 1100–9500 cm^−1^) are more intense than bands from pectin and lignin.

Our study demonstrated that vibrational spectroscopy methods, like FT-IR spectroscopy and imaging approaches coupled with HCA analysis, are suitable tools for ancient plant fibres analysis, however the obtained data might benefit from the subsequent verification with additional methods, for example conventional microscopic and staining techniques [53], chromatographic determination [54], fluorescence-based [55] and ELISA assays [56], and radioactive isotope labelling for the Solid-State NMR studies [57]. There are also numerous reports on the application of Raman and FT-Raman spectroscopy and imaging in this field [14,18,28,58,59], which is a complementary technique to the FT-IR spectroscopy, however, the limitation may be high fluorescence and sample heating. The attempt at using Raman microspectroscopy of hemp fibres was made during preliminary data collection with the use of two wavelength lasers: 532 nm and 780 nm. Unfortunately, in these particular samples, fluorescence phenomena did not allow recording adequate spectra. Although fluorescence might be significantly reduced by choosing the near infrared excitation wavelength, this method often causes resonance enhancement of respective sample components, which is inadvisable in case of detection of species at low concentrations or when constituent selectivity is important [60].

## 4. Materials and Methods

### 4.1. Sample Collection and Preparation

In January 2019, a new 97-cm long sediment core (SL-19) collected from lake Słone located in Poland (51°18′16″ N, 23°21′55″ E) was sampled in order to study in detail the lake microbiome, as well as microbiological processes of its recent (last 2000 years) history under the various forms of human impact. Immediately after collection, the core was wrapped in a non-transparent black film foil and transported to the laboratory, where it was sliced to 1 cm thick sub-samples, flash frozen in liquid nitrogen and kept at −80 °C until further analysis.

The analyses of several paleolimnological proxies of the SL-19 (e.g., pollen, Cladocera, LOI_550_, LOI_950_—data not shown) and independent age determination by ^14^C method enabled a precise correlation of the core with the previously published material, as well as pinpointing the layer of maximum disruption due to hemp retting—65 cm—which was sampled and examined in detail in this study (Table 2).

Hemp macrofossil material (fibre) was manually extracted from the sediment layer collected from the depth of 65 cm (layer of the highest content of *Cannabis sativa* pollen) under a dissecting microscope. About 40 fibres were placed in a sterile Petri dish and soaked (retted) overnight in 25 mL of distilled water at 4 °C (Slone_4) and 37 °C (Slone_37). Hemp fibres extracted from the sediments directly before the measurement were used as a control (Fresh).

### 4.2. FT-IR Microspectroscopy

Unprocessed and not subjected to any preparation prior to the analysis (apart from the overnight incubation) plant samples were placed on the reflective side of the aluminium-coated glass slides (DRLI, Deposition Research Lab Inc., St. Charles, MO, USA). Fibres were studied under the same conditions with the FT-IR spectroscope in transflectance mode (Nicolet 8700, Thermo Scientific, Madison, WI, USA) combined with the infrared microscope (Nicolet Continuµm, Thermo Scientific, Madison, WI, USA) equipped with ×15 objective. A liquid nitrogen cooled MCT (HgCdTe) detector for simultaneous acquisition of IR spectral data was utilized. Spectra were recorded in the range of 4000–600 cm^−1^ with 120 scans at 4 cm^−1^ resolution and aperture of 20 µm × 20 µm with an optimal signal-to-noise ratio. Three maps were collected for each sample: Fresh, Slone_4 and Slone_37. X and Y step size was 20 µm. Subsequent data analysis was performed with Omnic 12 (Thermo Fisher Scientific, Madison, WI, USA) and CytoSpec (ver. 2.00.01, Berlin, Germany). For the analysis, ten single spectra were recorded at various points of the investigated samples, averaged into one spectrum representative for a given sample and normalized to the band at 2918 cm^−1^ attributed to CH stretching. Baseline corrections were multipoint and applied at 3680, 2630, 1780, 1185, 910 and 650 cm^−1^. For each sample the characteristic peaks were marked and assigned to the vibrational bands. Ten spectra of each sample were subjected to absorbance intensity measurement. The intensities of bands at 2900, 1735, 1595 and 1105 cm^−1^ were measured above local baselines between 3700–2600, 1780–1485 and 1185–765 cm^−1^, respectively, and the results were collected in a spreadsheet. Ratios of the primary polysaccharides compared to the total organic material were calculated. The outputs were then subjected to statistical analysis using Statistica 13 (StatSoft Inc., Tulsa, OK, USA) software for calculation of arithmetic means and standard deviations (SD) of the intensities for each sample. Data was then expressed as a bar graph for samples relative content comparison. In order to assess the molecular changes in the studied samples, second order derivative spectra were calculated by the algorithm of Savitzky and Golay in 1730–1610, 1610–1490, and 1450–800 cm^−1^ ranges, including corresponding to amide I, II and III regions respectively. The hierarchical cluster analysis (HCA) was performed using the D-value distance (Pearson’s correlation coefficient) and Ward’s algorithm in CytoSpec (ver. 2.00.01, Berlin, Germany). As a result, dendrograms of similarity classes of spectral groups identified in hemp fibres were obtained. Then, three-cluster analysis was presented as a result with spatial distribution and spectral data distinction.

## 5. Conclusions

Infrared spectroscopy and imaging studies showed that microorganisms from ancient lake sediments were able to degrade polysaccharides in the remains of hemp bast fibres causing decrease in lignin and pectin content, while least affecting cellulose concentration. We demonstrated that FT-IR technique is suitable for the qualitative and semi-quantitative determination of the biochemical composition and homogeneity of spatial distribution within the ancient plant fibres. Our data strongly implicates usefulness of these techniques for assessment of the secondary structure of components of the plant material and their relative concentration. Simultaneous and non-invasive FT-IR microspectroscopy is highly beneficial in analysis of fragile ancient plant material.

## Figures and Tables

**Table 1:** Infrared band assignments for Fresh, Slone_4 and Slone_37 samples.

FT-IR Bands (Wavenumbers/cm^−1^)			Approximate Assignment of Vibrational Mode	Remarks [23,24,25]
Fresh	Slone_4	Slone_37		
3368	3382	3352	ν(OH) free [23]	cellulose, hemicellulose, lignin, pectin
2918	2918	2917	ν(CH) [24]	general organic content
2849	2850	2849	ν_s_(CH_2_) [24]	waxes
-	-	1736	ν C=O ester band [23,24]	pectin, waxes
-	1636	1633	ν(C=O) in plane, O-H [23]	amide I adsorbed water
1598	-	1597	ν(C=C) aromatic in-plane [24]	lignin
1512	1510	1511	Aromatic skeletal vibrations [26]	amide II
1461	1461	1461	δ(CH_2_), δ(COH) [14] τO-H [23]	lignin, cellulose, hemicellulose, pectins, waxes and fats
1424	1419	1421	δ(C-H) [24] νCOO [23] τ(HCH) τ(OCH) in-plane [3]	carboxylic acid, pectins, cellulose
1372	-	1377	δ(C-H) [20,23] δC-CH_3_ symmetrical [23]	lignin, cellulose
1263	1265	1261	δ(CH_2_) twisting	amide III
1148	-	1155	ν_as_(CC) ring breathing [25] τC-O-C [23]	cellulose, hemicellulose, pectin
-	1126	-	ν_as_(COC) glycosidic [14] νC-O [23,27]	cellulose, hemicellulose, pectin
1034	1034	1036	ν(CO), 1° alcohol [14]	cellulose, hemicellulose, pectin
894	-	-	ν_s_(C-O-C) in plane [23] Characteristic of β-links in cellulose [26]	cellulose, hemicellulose, pectin
814	714	-	δ(C–OH)ring [28]	cellulose

Vibrational modes assignment: stretching (ν), deformational (δ); bending (τ), and symmetrical (s) and asymmetrical (as).

**Table 2:** The results of age determination by the radiocarbon dating method.

Depth (cm)	Laboratory Code	^14^C Date [^14^C BP]	Calibrated Age—1σ Range (cal BCE/CE) 68.3% Probability	Calibrated Age—2σ Range (cal BCE/CE) 95.4% Probability	Dated Material
65	Poz-123833	1345 ± 30 BP	650 CE (55.4%) 680 CE 747 CE (12.9%) 758 CE	643 CE (68.3%) 705 CE 738 CE (27.1%) 774 CE	Terrestrial Plant Macrofossil —*Cannabis sativa* L.

## Data Availability

The data that support the findings of this study are available from the corresponding authors upon reasonable request.

## Sample Availability

Samples are not available.

## Appendix A

**Table A1:** Mean values with standard deviation of band absorbance intensities and ratios (R_1_=I_1595_/I_2900,_ R_2_=I_1595_/I_2900_ and R_3_=I_1735_/I_2900_) of Fresh, Slone_4 and Slone_37 samples.

Sample	Band/Ratio	Absorbance Intensity/a.u. ±SD
Fresh	2900 cm^−1^	0.171 ± 0.046
	1595 cm^−1^	0.189 ± 0.047
	1105 cm^−1^	0.104 ± 0.033
	1735 cm^−1^	0.046 ± 0.018
	R_1_=I_1595_/I_2900_	1.136 ± 0.224
	R_2_=I_1105_/I_2900_	0.628 ± 0.177
	R_3_=I_1735_/I_2900_	0.277 ± 0.118
Slone_4	2900 cm^−1^	0.197 ± 0.055
	1595 cm^−1^	0.192 ± 0.069
	1105 cm^−1^	0.145 ± 0.044
	1735 cm^−1^	0.019 ± 0.018
	R_1_=I_1595_/I_2900_	0.967 ± 0.158
	R_2_=I_1105_/I_2900_	0.752 ± 0.231
	R_3_=I_1735_/I_2900_	0.094 ± 0.057
Slone_37	2900 cm^−1^	0.220 ± 0.112
	1595 cm^−1^	0.185 ± 0.081
	1105 cm^−1^	0.138 ± 0.082
	1735 cm^−1^	0.045 ± 0.020
	R_1_=I_1595_/I_2900_	0.904 ± 0.206
	R_2_=I_1105_/I_2900_	0.631 ± 0.250
	R_3_=I_1735_/I_2900_	0.235 ± 0.131
